# Non‐operative management of bilateral contained thoracic esophageal perforation: a case report

**DOI:** 10.1186/s12893-021-01121-6

**Published:** 2021-03-12

**Authors:** Armin Amirian, Reza Shahriarirad, Parviz Mardani, Maryam Salimi

**Affiliations:** 1 Thoracic and Vascular Surgery Research Center, Shiraz University of Medical Sciences, Shiraz, Iran; 2 Student Research Committee, Shiraz University of Medical Sciences, Shiraz, Iran; 3 Department of Surgery, Shiraz University of Medical Sciences, Shiraz, Iran

**Keywords:** Esophageal perforation, Esophagus, Non‐operative management, Case report

## Abstract

**Background:**

Despite profound advances in conservative management of esophageal perforation, patients’ selection for this type of treatment requires expert clinical judgment. Surgical intervention has been historically introduced as the optimal management in multifocal ruptures.

**Case presentation:**

Here, we presented a 30-year-old man whose barium esophagogram confirmed bilateral perforations in the lower third of the esophagus contained in the mediastinum, and contrast drained back into the esophageal lumen. Concerning available contrast imaging studies and thoracic surgeons, conservative non-operative management was considered despite pneumomediastinum, a mild right-sided pleural effusion, and minimal leukocytosis. The patient was followed up for two months without any complications.

**Conclusions:**

Bilateral and multifocal esophageal perforations can be managed conservatively provided that the leaks are confined to the mediastinum and drain back to the esophageal lumen, and other criteria for conservative management are met.

## Background

Esophageal perforation, presenting as spontaneous esophageal rupture, is a life-threatening disease [1]. Clinical characteristics are correlated with the location and cause of the injury, alongside the interval of diagnosis and occurrence. Frequent clinical presentations of esophageal perforation contain dyspnea, epigastric pain, chest pain, dysphagia, subcutaneous emphysema, tachycardia, and tachypnea, fever. Diagnosis is often problematic due to diverse indecisive presentations, and it also usually mimics other disorders (e.g., peptic ulcer perforation, myocardial infarction, aortic aneurysm dissection, pneumonia, pancreatitis, or spontaneous pneumothorax) [2]. The gold standard in the diagnosis of esophageal perforation is contrast esophagography. However, despite the initial diagnosis accuracy rate of 30%, a mortality rate of 20–40% following severe respiratory failure is still observed among patients. Although the main treatment is surgical repair by primary suture with or without reinforcement, patients who do not progress to respiratory failure, sepsis, pneumoperitoneum, shock, pneumothorax, or extensive mediastinal emphysema can be managed conservatively following a 7 to 14 days broad-spectrum antibiotic regimen along with total parenteral nutrition [3, 4]. There are several non-operative management reports, mostly in unilateral and cervical esophageal perforation, while perforation of the abdominal or thoracic esophagus can lead to a relative dilemma in choosing non-operative management [5, 6]. Here we present a case of bilateral contained thoracic esophageal perforation that was managed successfully with conservative treatment.

## Case presentation

**Fig. 1:**
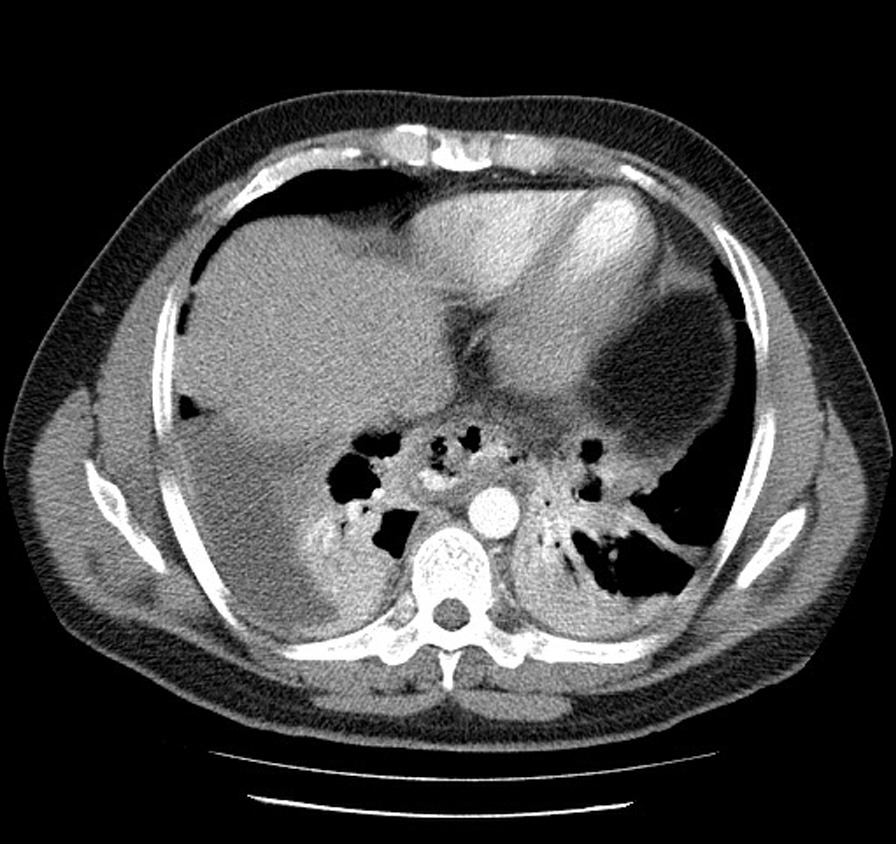
Computed tomography demonstrating pneumomediastinum and a mild right-sided pleural effusion due to esophageal perforation

A 30-year-old man came to our institution with a 5-day history of severe epigastric pain and odynophagia following the ingestion of a hot soup and concomitant forceful vomiting. He had a history of intermittent heartburn and dyspepsia during last year treated with proton pump inhibitors. There was no history of alcohol or drug abuse. Physical examination was normal except for mild tachypnea (respiratory rate: 20), and laboratory investigation showed leukocytosis, with a white cell count of 14,000 in each cubic millimeter (reference range, 3500–10,500). Computed tomography (CT) revealed pneumomediastinum and a mild right-sided pleural effusion (Fig. 1).

**Fig. 2:**
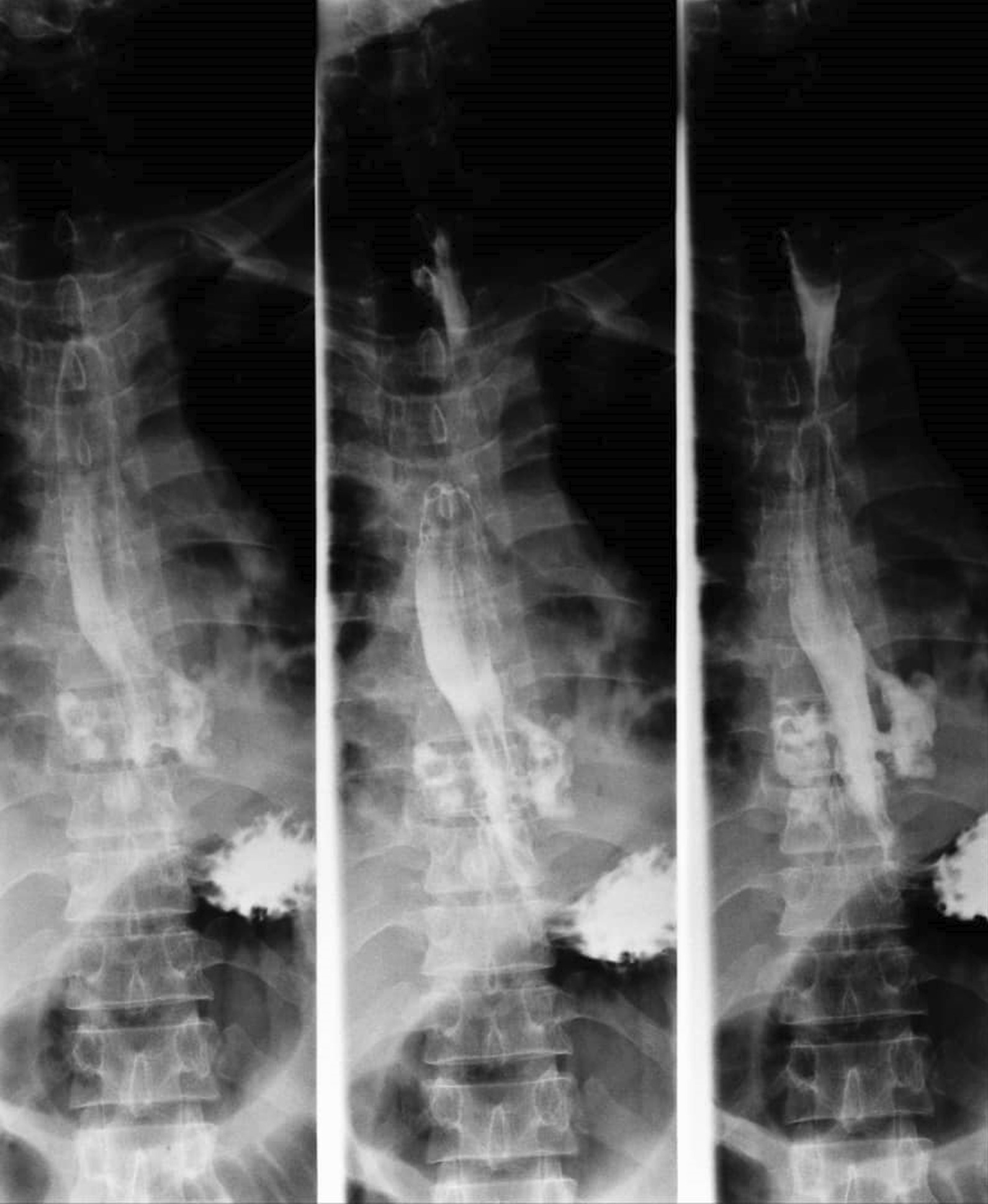
Barium esophagogram demonstrating bilateral perforations in the lower third of the esophagus

A thin barium esophagogram confirmed bilateral perforations in the lower third of the esophagus, contained in the mediastinum, and contrast drained back into the esophageal lumen (Fig. 2).

The patient was admitted to the intensive care unit (ICU) with large-bore intravenous access, was withheld from food and fluids, and managed by broad-spectrum intravenous antibiotics and total parenteral nutrition. Adequate analgesia was administrated to control pain and discomfort. Vital signs were stable, and the white-cell count decreased to 8,000 per microliter in 6 days. After ten days, repeated esophagogram showed complete resolution of previous perforations. A surgical diet was started and changed to a regular diet after two days. The patient was discharged after tolerating the diet and visited the outpatient clinic after 2 weeks and two months with no further complaints and also, no signs or symptoms of stricture was observed in the patient during his 6-month follow-up.

## Discussion

Esophageal rupture is a crucial disorder due to its controversial diagnosis and management, accompanied by a 7–33% mortality rate [7]. Furthermore, thoracic esophageal rapture has the utmost mortality based on the injury site [3]. The standard treatment contains initial repairing of the perforation location along with purging of a distal hindrance. However, non-operative therapy is suitable in particular well-defined conditions such as late detected stable patients or non-obstructive neoplasms. [8]

Lampridis et al. claimed that in patients who suffer from ruptures in the lower third of the esophagus, optimal management is reached by a thoracotomy in the right side of the sixth intercostal area following thoracotomy in the left side of the seventh intercostal space [5]. Depending on the availability of a skilled surgeon and accessibility to contrast imaging studies, in case the patients’ condition or vital signs deteriorates, a decision was made to manage him non-operatively and conservatively along with careful monitoring in ICU. Our patient selection for non-operative management was consistent with the criteria described by Altorjay, including delayed diagnosis with a contained leak within the mediastinum, drainage into the esophageal lumen, which is demonstrated by contrast imaging, absence of the availability of contrast imaging along with symptoms, and signs of septicemia, as well as an experienced thoracic surgeon [9].

Conversely, the bilateral perforation was a troublesome issue. According to favorable opinion, several studies have reported that if treatment is postponed for over 24 h after the injury, the treatment modality does not affect the outcome, and the majority of cases can be managed through non-operative treatment [3, 7]; This was the case in our report, based on the five-day interval between occurrence and diagnosis.

Endoluminal vacuum sponge therapy, which was introduced as an alternative method for more invasive surgical repair procedures, seems to have its specific limitations. The risk of pleural and peritoneal contamination and sponge rupture during removal procedures should be considered in this method, along with fatal sepsis and failed treatment, which has been reported in cases with large perforation [10]. Therefore, this therapeutic option was not considered in our current case, which presented with bilateral perforation. Furthermore, unfortunately, interventions such as endoscopic stenting and endosponge are not readily available in our country. Also, lack of expertise with these equipments persists, resulting in the absence of clinical practice implication of these methods in our center.

Regarding endoscopic evaluation, since there was no evidence of foreign bodies in esophagus based on radiologic findings and conservative management was chosen, an endoscopic intervention was not safe because it may lead to further iatrogenic injury of the esophagus and extending minor perforations. The patient was clinically stable and fulfilled the criteria for nonoperative management (hemodynamic stability, absence of clinical sepsis, confined leakage in mediastinum), so drainage and operation were not performed.

To sum up our report, nowadays, concerning surgical complications (e.g., stricture formations and prolonged hospital stay), there is an increasing trend for shifting from operative techniques to more conservative, non-operative, less invasive methods. Bilateral and multifocal esophageal perforations can be managed conservatively provided that the leaks are confined to the mediastinum and drained back to the esophageal lumen, and other criteria for conservative management are met. Surgeons worldwide should be aware of these management options to help their decision-making process in whether to operate or not, to offer the most beneficial therapeutic method for their patients.

## Data Availability

All relevant data regarding this case report has been reported in the manuscript. Please contact the corresponding author for any further information.

## Abbreviations

CT: Computed tomography
ICU: Intensive care unit
